# Immunotherapy for non-small cell lung cancers: biomarkers for predicting responses and strategies to overcome resistance

**DOI:** 10.1186/s12885-018-4990-5

**Published:** 2018-11-08

**Authors:** Xingxiang Pu, Lin Wu, Dan Su, Weimin Mao, Bingliang Fang

**Affiliations:** 1 Department of Thoracic and Cardiovascular Surgery, The University of Texas MD Anderson Cancer Center, Houston, TX 77030 USA; 2 Department of Thoracic Medical Oncology, Hunan Cancer Hospital/the affiliated Cancer Hospital of Xiangya school of Medicine, Central South University, 283 Tongzipo Road, Yuelu District, Changsha, 410013 Hunan China; 3 Department of Pathology, Zhejiang Cancer Hospital, 38 Guanji Road, Banshan Bridge, Hangzhou, 310022 Zejiang China; 4 Department of Thoracic Surgery, Zhejiang Cancer Hospital, 38 Guanji Road, Banshan Bridge, Hangzhou, 310022 Zejiang China

**Keywords:** Immune checkpoint inhibitors, PD-1, PD-L1, Predictive biomarkers, Resistance

## Abstract

Recent breakthroughs in targeted therapy and immunotherapy have revolutionized the treatment of lung cancer, the leading cause of cancer-related deaths in the United States and worldwide. Here we provide an overview of recent progress in immune checkpoint blockade therapy for treatment of non-small cell lung cancer (NSCLC), and discuss biomarkers associated with the treatment responses, mechanisms underlying resistance and strategies to overcome resistance. The success of immune checkpoint blockade therapies is driven by immunogenicity of tumor cells, which is associated with mutation burden and neoantigen burden in cancers. Lymphocyte infiltration in cancer tissues and interferon-γ–induced PD-L1 expression in tumor microenvironments may serve as surrogate biomarkers for adaptive immune resistance and likelihood of responses to immune checkpoint blockade therapy. In contrast, weak immunogenicity of, and/or impaired antigen presentation in, tumor cells are primary causes of resistance to these therapies. Thus, approaches that increase immunogenicity of cancer cells and/or enhance immune cell recruitment to cancer sites will likely overcome resistance to immunotherapy.

## Introduction

Recent breakthroughs in immunotherapy for cancer have changed clinical practice in the treatment of lung cancer, a deadly disease that each year causes about 155,000 deaths in the United States and approximately 1.6 million deaths worldwide [1, 2]. Since 2015, four immune checkpoint inhibitors (ICIs), anti–PD-1 antibodies nivolumab [3, 4] and pembrolizumab [5, 6] and anti–PD-L1 antibody atezolizumab [7] and durvalumab [8, 9], were approved for treatment of non–small cell lung carcinoma (NSCLC) by the United States Food and Drug Administration (FDA). Nivolumab [10] and atezolizumab [11] were approved in 2015 and 2016, respectively, as second-line therapies for patients with advanced NSCLC that progressed after or during platinum-based chemotherapy. Pembrolizumab was approved in 2015 as a second-line therapy for patients with advanced NSCLC with PD-L1 expression of ≥1% [12]. In 2016, pembrolizumab was approved as a first-line therapy for NSCLC with PD-L1 expression of ≥50% in tumor tissues and for advanced NSCLC which has PD-L1 expression of ≥1% and has disease progression on or after platinum-containing chemotherapy [12]. More recently, pembrolizumab in combination with pemetrexed and carboplatin was approved by the FDA as first-line therapy for NSCLC [13], while durvalumab was approved for treatment of patients with unresectable stage III NSCLC whose cancer have not progressed following treatment with chemotherapy and radiation [8, 9].

Clinical trials have revealed that using anti-PD immunotherapy for patients with advanced NSCLC led to improved clinical outcomes, including improved survival rates, prolonged duration of response, and reduced treatment-related adverse effects [14]. However, although anti–PD-1 and anti–PD-L1 therapies have shown unprecedented durable responses in some NSCLC patients, emerging data from clinical trials with anti-PD therapy also revealed that only about 15–25% of NSCLC patients responded to immune checkpoint blockage therapy [3–5, 7], and most had primary resistance. Thus, predictive biomarkers need to be identified for patient stratification in order to maximize the therapeutic benefit of immune checkpoint blockade therapy; furthermore, approaches to overcome resistance to this anticancer immunotherapy are highly desirable in order to broaden the patient populations who can benefit from this therapy. This review discusses recent progress in translational and clinical investigation of immune checkpoint blockade therapy for lung cancer and factors associated with the treatment responses. We first review the currently available immune checkpoint blockade therapies for treatment of non-small cell lung cancer (NSCLC), and then discuss biomarkers associated with the treatment responses, and analyze possible mechanisms underlying resistance and strategies to overcome resistance.

### Clinical responses to immune checkpoint inhibitors in NSCLC

Since 2015, four PD-1/PD-L1 inhibitors (nivolumab, pembrolizumab, atezolizuma and durvalumab) have been approved by the FDA as second-line therapy and/or first-line therapy for NSCLC. In addition, anti-PD-L1 antibody avelumab [15] is being evaluated extensively in clinical trials for treatment of NSCLC.

Nivolumab is a human antibody (IgG4) specific for human PD-1. It binds PD-1 with high affinity and prevents interaction of PD-1 with its ligands PD-L1 or PD-L2, thereby enhancing tumor antigen-specific T cell proliferation [16]. The human IgG4 immunoglobulin subtype interacts poorly with Fc receptors (FcγRII and FcγRIII) and complement [17], and thus causing minimal antibody-dependent cell-mediated cytotoxicity and/or complement-induced cytotoxicity to the T cells to be activated. In phase 1 and 2 trials, nivolumab showed durable antitumor activity and a cumulative response rate of about 18% in all NSCLC subtypes [18–20]. In two phase 3 studies comparing the efficacies of nivolumab versus docetaxel in advanced squamous (CheckMate 017) [4] and nonsquamous (CheckMate 057) [3] NSCLC that were resistant to platinum-based therapy, nivolumab was found to be significantly better than docetaxel in response rate, overall survival, and progression-free survival, regardless of intratumoral PD-L1 expression levels. The overall response rate was 19–20% in the nivolumab treated group versus 9–12% in docetaxel treated group (Table 1). Based on those results, nivolumab was approved by the FDA in 2015 as a second-line monotherapy for advanced squamous cell and non-squamous cell NSCLC.

**Table 1:** Clinical trials on immune checkpoint inhibitors in non–small cell lung cancer

Name of trial	Phase	Histology/ line of treatment	Randomization	No. Cases	First end point results	ORR (RECIST)	Effect of PD-L1 expression
CheckMate 017	III	SqNSCLC/ second	Nivolumab at 3 mg/kg vs. docetaxel at 75 mg/m^2^	272	Significant improvement in OS for patients receiving nivolumab compared with docetaxel (median, 9.2 vs. 6.0 mo; HR, 0.59; *p* < .001).	Response rate was 20% with nivolumab vs. 9% with docetaxel (*P* = 0.008)	PD-L1 expression was neither prognostic nor predictive for efficacy end points
CheckMate 057	III	Non-SqNSCLC/second	Nivolumab at 3 mg/kg vs. docetaxel at 75 mg/m^2^	582	Significant improvement in OS for patients receiving nivolumab compared with docetaxel (median 12.2 vs. 9.4 mo; HR, 0.73; *p* = .002).	Response rate was 19% with nivolumab vs. 12% with docetaxel (*P* = 0.02)	PD-L1 expression was associated with even greater efficacy at all expression levels (≥1%, ≥5%, and ≥ 10%).
KEYNOTE 010	II/III	NSCLC PD-L1-positive tumors (PS ≥ 1%)/second	Pembrolizumab at 2 mg/kg or 10 mg/kg vs. docetaxel 75 mg/m^2^	1034	Significant improvement in OS for pembrolizumab at 2 mg/kg (median 10.4 vs. 8.5 mo; HR, 0.71; *p* = .0008) and pembrolizumab at 10 mg/kg (median, 12.7 vs. 8.5 mo; HR, 0.61; *p* < .001) compared with docetaxel	Response rate was 18% with pembrolizumab (2 mg and 10 mg vs. 9% with docetaxel (*P* = 0.0005 and 0.0002)	Pembrolizumab efficacy was greater in patients with tumor PS ≥50%
KEYNOTE 024	III	NSCLC, PD-L1-positive tumors (PS ≥50%), no sensitizing mutation of EGFR or translocation of ALK/first	Pembrolizumab at fixed dose of 200 mg or platinum-based chemotherapy	305	Significant improvement in PFS for patients receiving pembrolizumab compared with chemotherapy (median 10.3 vs. 6.0 mo; HR, 0.5; *p* < .00001).	Response rate was 44.8% with pembrolizumab vs. 27.8% with chemotherapy	All patients, PD-L1 expression on at least 50% of tumor cells
POPLAR	II	NSCLC/ second	Atezolizumab 1200 mg vs. docetaxel 75 mg/m^2^	287	Significant improvement in OS for patients receiving atezolizumab compared with docetaxel (median, 12.6 vs. 9.7 mo; HR, 0.73; *P* = .04)	Objective responses with atezolizumab were durable, with a median duration of 14·3 months (95% CI 11·6–non-estimable) compared with 7·2 months (5·6–12·5) for docetaxel	As with OS, PFS and ORR tended to show increased atezolizumab benefit with increasing PD-L1 expression.
OAK	III	NSCLC/ second	Atezolizumab at 1200 mg vs. docetaxelat 75 mg/m^2^	850	Significant improvement in OS for patients receiving atezolizumab compared with docetaxel (median 13.8 vs. 9.6 mo; HR, 0.73; *P* = .0003).	For ITT population, response rate was 14% with atezolizumab vs. 13% with docetaxel	Overall survival was improved regardless of PD-L1 expression levels. Patients with tumors expressing high levels of PD-L1 (TC3 or IC3) derived the greatest benefit from atezolizumab
PACIFIC	III	Stage III NSCLC with no disease progression after ≥2 cycles of chemoradiotherapy/second	Durvalumab at 10 mg/kg vs. placebo	709	Significant improvement in PFS and OS for patients with durvalumab vs. with placebo (PFS median 17.2 vs. 5.6 mo; HR, 0.51, *P* < 0.001; HR for OS =0.68, *P* = 0,0025);	Response rate was 28% with durvalumab vs. 16% with placebo	PFS and OS benefits with durvalumab were observed in all subgroups, including PD-L1 expression ≥25% or < 25%

*Abbreviations*: *OS* overall survival, *NSCLC* non–small cell lung cancer, *Sq* squamous, *HR* hazard ratio, *ORR* objective response rate, *ITT* Intent to treat, *PD-1*, programmed cell death protein-1, *PD-L1* programmed cell death ligand-1, *PS* proportion score

Pembrolizumab (KEYTRUDA) is a humanized IgG4 monoclonal antibody specific for human PD-1. In a randomized phase 2 and 3 trial (KEYNOTE-010) with 1034 NSCLC patients who were previously treated with chemotherapy and were PD-L1–positive in tumor cells based on immunohistochemical analysis (≥1%) [21] (Table 1), patients were randomly assigned to three arms: pembrolizumab at 2 mg/kg, pembrolizumab at 10 mg/kg, and docetaxel at 75 mg/m^2^. The results showed that, among patients with at least 50% of tumor cells expressing PD-L1, overall survival (OS) and progression-free survival (PFS) were significantly longer in the group treated with pembrolizumab at 2 mg/kg than the group treated with docetaxel (median OS was 14.9 months vs 8.2 months, respectively; median PFS 5.0 months vs 4.1 months, respectively) and with pembrolizumab at 10 mg/kg than with docetaxel (median OS was 17.3 months vs 8.2 months, respectively; median PFS 5.2 months vs 4.1 months, respectively) [21]. In another phase 3 trial (KEYNOTE-024) with 305 advanced NSCLC patients who were not previously treated and had no sensitizing mutation for target therapies in their tumors but had at least 50% PD-L1^+^ tumor cells, patients were randomly assigned to the treatment with either pembrolizumab (200 mg every 3 weeks) or platinum-based chemotherapy [22]. The results revealed that both PFS and estimated OS at 6 months were significantly improved in pembrolizumab treated group than in the chemotherapy group. The response rate was about 45% in the pembrolizumab group vs approximately 28% in the chemotherapy group. Those results led to pembrolizumab’s approval as second-line therapy for metastatic NSCLC with PD-L1 expression of ≥1% and first-line therapy for NSCLC with expression of PD-L1 of ≥50%.

Atezolizumab is an anti-PD-L1 antibody that previously approved by the FDA for the treatment of urothelial carcinoma that progresses after platinum-based chemotherapy. Atezolizumab was recently approved as a second-line therapy for patients with metastatic NSCLC based on two international trials (OAK and POPLAR, Table 1) with a total of 1137 NSCLC patients, which demonstrated consistent results in efficacy and safety atezolizumab in treatment of NSCLC [7, 23]. In comparison with docetaxel, treatment with atezolizumab led to a 2.9 ~ 4.2 month improvement in OS in these two trials. The median OS was about 13 months in the atezolizumab treated group compared with about 9.6 months in the docetaxel treated group [7, 23]. The improvement in OS was associated with increased expression of PD-L1 in tumor cells and increased tumor-infiltrating immune cells [23].

Durvalumab is a PD-L1 specific human IgG1 monoclonal antibody [24] that contains three point mutations in the constant domain for minimized binding to complement and Fc receptors [25]. Durvalumab was recently approved for treatment of patients with locally advanced or metastatic urothelial carcinoma who have disease progression during or following platinum-containing chemotherapy [26]. In a phase III trial (PACIFIC) of 709 stage III NSCLC patients who did not have disease progression after two or more cycles of platinum-based chemoradiotherapy, durvalumab was found to have significantly better PFS, response rate, median time to death or distant metastasis, and OS when compared with placebo [8, 9], which led to FDA’s approval of durvalumab for treatment of uresectable stage III NSCLC whose disease has not progressed following concurrent platinum-based chemotherapy and radiation therapy. The PFS and OS benefits with durvalumab were observed irrespective of PD-L1 expression before chemoradiotherapy based the stratification of PD-L1 ≥ 25% or < 25%. In another trial (ATLANTIC) with 444 NSCLC patients enrolled in three cohorts, it was found that the proportion of patients with EGFR^−^/ALK^−^ NSCLC achieving a response was higher than that with EGFR^+^/ALK^+^ NSCLC, nevertheless durvalumab activity was observed in patients with EGFR^+^ NSCLC whose tumor has ≥25% PD-L1 expression [27].

### Predictive biomarkers associated with response to ICIs

Multiple biomarkers have emerged as being associated with treatment responses to immune checkpoint blockade therapies, including tumor mutational load [28, 29], DNA mismatch repair deficiency [30, 31], composition of gut microbiome [32, 33], intensity of CD8+ cell infiltration [34] and intratumoral PD-L1 expression [35].

The presence of tumor-specific antigens and the interaction of immune cells with tumor antigens are the two basic principles of cancer immunology. Tumor antigens can derive from mutant proteins, overexpressed or dysregulated embryonic proteins, and oncogenic viral proteins. Increased nonsynonymous mutation burden in tumor tissues was expected to increase neoantigens in tumor, leading to stronger immune response against cancer cells. Indeed, clinical trials in lung cancer and melanoma have shown that high tumor mutation burden (TMB) was significantly associated with better objective response, durable clinical benefit, and prolonged progression-free survival for patients treated with ICIs [28, 29]. Analysis on data of ICI therapy available in literature for 27 cancer types revealed a significant correlation between the TMB and the objective response rate of anti-PD-1/PD-L1 therapies [36]. In a randomized phase III trial with stage IV or recurrent NSCLC, nivolumab as first-line therapy was found not superior to chemotherapy in PFS or response rate in patients whose tumor had PD-L1 expression of ≥5%. However, nivolumab was found to have higher response rate and longer PFS than chemotherapy among patients with high TMB [37]. Nevertheless, in another phase III trial with stage IV or recurrent NSCLC that was not previously treated with chemotherapy, no significant difference in PFS was found between nivolumab monotherapy and chemotherapy in patients with high TMB [38]. However, TMB was found to be strongly associated with efficacy of ICI combination therapy and was independent of PD-L1 expression [38, 39]. High TMB predicted better objective response, durable benefit and longer PFS in patients treated with nivolumab plus ipilimumab when compared with chemotherapy, of regardless PD-L1 expression. In patients with a low TMB, however, nivolumab plus ipilimumab didn’t provide a benefit over chemotherapy, suggesting that ICI combination therapy alone is insufficient to overcome the resistance caused by low immunogenicity in tumors.

Lung cancer is known to have high TMB when compared with other cancers [40], presumably because lung is directly exposed to mutagens present in tobacco smoke. The average mutation frequency in lung cancer is more than 10-fold higher in smokers than in never-smokers [41]. In fact, molecular smoking signature is significantly associated with improved PFS in patients treated with pembrolizumab, although self-reported smoking history did not significantly associate with benefit of pembrolizumab treatment [28]. In addition to direct exposure to mutagens, driver mutations in genes involved in DNA replication and repair pathways drastically impact on the scale of mutation load [42]. For example, cancers with loss-of-function mutations in genes required for DNA mismatch repair, such as *MLH1, MSH2, MSH6*, and *PMS2*, are known to have increased microsatellite instability and to have 100- to 600-fold increases in gene mutation rates [43, 44]. Most solid tumors contain, on average, 60 to 140 nonsynonymous mutations in their genome [40, 45], whereas cancers with mismatch repair deficiency contain, on average, 1400 to 1600 nonsynonymous mutations [31]. Tumors with DNA mismatch repair deficiencies showed greater densities of CD8+ tumor-infiltrating lymphocytes (TILs) and higher PD-L1 expression, and improved response rates and higher survival rates were achieved when patients with these tumors were treated with immune checkpoint inhibitors [30, 31]. DNA mismatch repair deficiency and/or microsatellite instability are detected in about 10% to 15% of colorectal, ovarian, gastric, and endometrial cancers [46–48]. Nevertheless, DNA mismatch repair deficiency or microsatellite instability is detected in less than 1% of NSCLC [49, 50].

High neoantigen burden in tumors are expected to trigger an anticancer immune response, recruiting immune cells to cancer sites and leading to increased levels of TILs, especially effector CD8+ T cells, and immune regulatory cells, such as helper T cells, Tregs, dendritic cells, and macrophages at the cancer site. Activation of T lymphocytes by tumor antigen ultimately leads to production and secretion of IFNγ, a cytokine that stimulates TIL proliferation and differentiation, thereby enhancing TIL’s effector functions. Paradoxically, IFNγ also trigger TIL apoptosis by inducing PDL1 expression in the tumor microenvironment [51–53], which causes to a negative-feedback that eventually inhibits antitumor immunity.

PD-L1 is not expressed in most normal tissues. However, its expression can be induced by some cancer drivers [54, 55] or by IFNγ [56, 57]. IFNγ-induced PD-L1 expression has a unique histopathological pattern, which is usually focal rather than diffuse and is expressed in cells adjacent to TILs, as observed in most human cancers [51, 58, 59]. Therefore, both increased TIL levels and increased expression of PD-L1 in the tumor tissues can serve as surrogate biomarkers of immunogenicity of cancer cells and interactions between cancer cells and immune cells or of the presence of adaptive immune resistance [58, 60, 61]. It has been proposed that, based on the presence or absence of PD-L1 expression and TILs, the tumor microenvironment can be classified into four groups [62, 63]: 1) TIL and PD-L1 double-positive, suggesting the presence of adaptive immune resistance; tumor will likely benefit from PD-L1/PD-1 blockade therapy; 2) TIL and PD-L1 double-negative, indicating immune ignorance or lack of detectable immune reaction; tumor will likely not to benefit from ICI therapy; 3) TIL-negative but PD-L1–positive; indicating that PD-L1 expression in cancers is independent of IFNγ but is intrinsic through oncogenic signaling; tumor may not respond to immune checkpoint inhibitors; and 4) TIL-positive but PD-L1–negative, indicating that other immune-suppressive mechanisms may mediate immune tolerance; targeting alternative immune suppressive pathways will be required to restore anticancer immunity.

This classification of the tumor microenvironment provides some insight to the discordant results observed in clinics and suggests that PD-L1 expression alone may not completely predict the response to immune checkpoint blockade therapy. Higher levels of PD-L1 expression in tumor tissues were found to have significantly better response rates and better survival in some ICI clinical trials, such as KEYNOTE-001 [5], CheckMate 057 [3], and POPLAR [23]. However, expression of PD-L1 was neither prognostic nor predictive of clinic benefit in CheckMate 017 [4]. In addition, intratumoral heterogeneity of neoantigens [64], different methods (distinct immunohistochemistry antibody clones, staining methods, and scoring systems), and different cutoff values in clinical evaluation of PD-L1 may have also led to discordant results [65].

### Lymphocytes reactive to neoantigens

Evidence has shown that intensive lymphocytes infiltrations in tumor stroma and/or intraepithelial tumor nest are associated with better prognosis in lung cancer [66–69]. For example, a study with more than 1500 cases of resectable NSCLC patients has showed that about 10% of NSCLC patients had intense lymphocytic infiltration in their tumors and this subset of patients had improved overall survival than the patients with nonintense tumor lymphocyte infiltration [68]. In particular, high density of CD8^+^ and/or CD4^+^ T cells in tumor stroma were independent favorable prognostic factors for NSCLC [66, 67, 69]. More recently, it has been shown that a subset of memory T cells, designated as tissue resident memory T cells (T_RM_), resident in tissues and do not recirculate via bloodstream. The majority of these T_RM_ cells express CD69 and CD103. Higher density of T_RM_ cells in tumors was recently reported to be predictive of a better survival outcome in lung cancer [70–72]. CD8^+^CD103^+^ TIL freshly isolated from NSCLC specimens often express both PD-1 and TIM-3. This TIL subset is found to have increased activation-induced cell death and is capable of mediating specific cytolytic activity against autologous tumor cells when PD-1/PDL1 interaction was blocked [70, 71].

Evidence has shown that naïve T cells are more effective than memory T cells and that central memory T cells (T_CM_) are more effective than effector memory T cells (T_EM_) in adoptive cellular therapy for cancers [73–75]. Analysis on a subpopulation of TILs in melanoma showed that tumor-reactive CD8^+^ T cells were largely derived from CD8^+^PD-1^+^ T cells, even though the level of PD-1 expression on CD8^+^ tumor-specific TILs decreased during culture with IL-2 [76, 77]. PD-1 overexpression is frequently detected in CD8^+^ T cells freshly isolated from melanoma, followed by TIM-3, 4-1BB, and LAG-3 [77]. Moreover substantial coexpression of these four receptors was detected on a subset of CD8^+^ TILs. Expression of TIM-3, LAG-3, and 4-1BB was almost exclusively present on PD-1^+^ cells. Up-regulation of multiple inhibitory receptors in CD8+ cells was observed in chronic antigen stimulation, including chronical infection, and is known to be associated with T-cell exhaustion [78, 79]. Interestingly, CD8^+^PD-1^+^ T cells isolated from the peripheral blood of melanoma patients are also neoantigen-reactive [80]. Tumor antigen (including mutated neoantigens, cancer germline antigens, and viral antigens)-specific T cells can be obtained from both tumor tissues and autologous peripheral blood from CD8^+^PD-1^+^ cell populations (at frequencies of about 0.4–0.002% in blood) [80–82]. The tumor antigen–specific CD4^+^ or CD8^+^ TILs targeting mutant ERBB2IP and KRAS have been shown to have high anticancer activity in patients [83, 84]. Of interest, the tumor-antigen specificities and TCR repertoires of the CD8^+^PD-1^+^ cells from peripheral blood and tumor tissues are similar [80], suggesting that the circulating CD8^+^PD-1^+^ T cells might be a novel noninvasive approach of adoptive cell therapy with neoantigen-reactive lymphocytes, or serve as a surrogate biomarker for adaptive immune resistance.

### Resistance to immune checkpoint inhibitors

The mechanisms of resistance to immune checkpoint blockade therapy are not yet well characterized but likely involve multiple factors. A recent study showed that abnormal gut microbiome composition due to the use of antibiotics before immune therapy inhibited the clinical benefit of immune checkpoint blockade therapy in cancer patients [33]. Several oncogenes and tumor suppressor genes have been reported to affect efficacy of ICI therapy [85–87]. Also, the presence of parallel immune inhibitory pathways and the loss of antigen presentation in cancer cells can be a common mechanism of resistance.

Activating mutations in the *EGFR* gene are found in approximately 10–17% of lung adenocarcinoma in Caucasians and in approximately 30–65% of lung adenocarcinoma patients in Asia [88]. Clinical trials with nivolumab [3], pembrolizumab [21], atezolizumab [7], and durvalumab [8] showed no significant survival benefit in *EGFR*-mutant patients treated with these ICIs. A meta-analysis on data from randomized trials of ICIs in NSCLC also found that no significant improvement in OS in the *EGFR* mutant subgroup treated with ICIs, although prolonged OS was observed in whole study populations and in the *EGFR* wild-type subgroup when compared with patients treated with docetaxel [86]. It is not clear whether the *EGFR*-mutant cancers have lower TMB than *EGFR*-wild type tumors. Intriguingly, treatment with ICIs was found to result in accelerated tumor growth and clinical deterioration in a subset of patients when compared with pretherapy. Genomic alterations in the *MDM2/MDM4* and *EGFR* genes were found to be correlated with this “hyperprogressor” phenotype [87]. Genomic alterations in the *STK11* gene are another factor reported to cause ICI resistance in NSCLC [85]. In lung adenocarcinoma patients with KRAS mutations, co-mutations in *STK11* were associated with inferior clinical outcome following PD-1 blockade therapy. *STK11* mutations is significantly enriched among tumors with intermediate/high TMB and negative PD-L1 expression. Moreover, knockout of *Stk11* resulted in resistance to PD-1 blockade therapy in a *Kras*-mutant syngeneic mouse model [85], demonstrating *STK11* mutations are a causal factor of resistance to ICIs.

Presence of parallel immune inhibitory pathways has been extensively investigated as the causal factors in ICI resistance. For example, indoleamine-2,3-dioxygenase (IDO), a metabolic enzyme that catalyzes the rate-limiting step of tryptophan degradation, has been reported to play a critical role in resistance to immunotherapy targeting CTLA-4 [89]. Induced by inflammatory signals such as prostaglandins, IFNγ, and tumor necrosis factor alpha (TNF-α) [90], IDO functions as a key mediator in activating and maintaining the immune suppressive function of Treg cells [91]. Coexpression of multiple T-cell inhibitory receptors (TCIRs), including PD-1, CTLA4, TIM3, and LAG3, in activated and/or exhausted T cells, suggested that parallel inhibitory pathways may mediate resistance to ICI therapy targeting a single TCIR and that simultaneous inhibition of multiple TCIRs may be required to improve therapeutic efficacy. Upregulation of TIM3 was found in PD-1 antibody bound T cells in both murine models of lung adenocarcinoma and in lung cancer patients with adaptive resistance to anti-PD-1 therapy [92]. Sequential PD-1 and TIM3 blockade prolonged survival in a mouse tumor model, indicating potential benefit of anti-PD-1 and anti-TIM3 combination therapy. Prolonged interferon signaling was reported to augment expression of interferon-stimulated genes, including multiple TCIRs and their ligands, leading to resistance to the ICI [93]. Impaired human leukocyte antigen (HLA) class I antigen processing and presentation due to homologous loss or down-regulation of B2M has also been found as a mechanism of acquired resistance to ICIs in lung cancer patients [94, 95]. CRISPR-mediated knock-out of *B2m* in an immunocompetent lung cancer mouse model conferred resistance to PD-1 blockade in vivo [94], demonstrating the causal relationship between loss of B2m and resistance to ICIs.

Because ICIs primarily target the immunosuppressive signals at cancer sites by locoregional blockade of negative feedbacks induced by inhibitory receptors [59], the absence of CTLs at tumor tissues, as observed in most cancer patients [58, 63], could be a major barriers for ICI therapy [35, 59]. In contrast, inflammatory signals are known to strongly augment activated T-cell homing to region of infection [96, 97]. It has been reported that presence of local infection and/or expression of foreign genes, T cell activations were induced by even very weak interactions between T-cell receptors and their ligands, resulting in rapid proliferation and amplification of immune effector and memory cells [98]. Thus, we hypothesized that resistance to ICI therapy caused by low immunogenicity of cancer cells or lack of immune cell infiltration at cancer sites might be overcome by inducing lymphocyte infiltration at cancer sites through installation of locoregional inflammatory signals.

To test this hypothesis, we investigated the effects of anti-PD therapy in combination with locoregional adenovirotherapy in the syngeneic lung cancer model M109. We found that the M109 tumor does not express PD-L1, does not have CD8^+^ or CD4^+^ lymphocyte infiltration in cancer tissues, and is resistant to both anti-PD-1 and anti–PD-L1 treatments. Intratumoral administration of an oncolytic adenovirus led to dramatic intratumoral infiltration of both CD4^+^ and CD8^+^ lymphocytes and sensitized the M109 tumor to the treatment of anti–PD-1 or anti–PD-L1 antibodies [99]. This result provided a proof-of-concept evidence that resistance ICIs in lung cancer can be overcome by locoregional virotherapies. A similar result was observed in a clinical trial of combination therapy of pembrolizumab with talimogene laherparepvec, a modified herpes simplex virus type 1 that selectively replicates in tumors and expresses granulocyte-macrophage colony-stimulating factor (GM-CSF), in patients with advanced melanoma [100]. IFNγ gene expression in tumor cells after talimogene laherparepvec treatment, increased CD8^+^ T cells and elevated PD-L1 protein expression were detected in cancers of the patients who responded to the combination therapy. In addition, baseline CD8^+^ T-cell infiltration or baseline IFNγ signature were not associated with the response to the combination therapy, suggesting that locoregional virotherapy may overcome primary resistance to ICI therapy by modulating the tumor immune microenvironment.

Promoting lymphocyte infiltration in tumors by other approaches, such as by targeted delivery of LIGHT (TNFSF14) gene to the tumor [101] or by targeted type I IFN activation through peritumoral injections of immunostimulatory RNA (poly:IC) [102], has also been shown to overcome resistance to anti–PD-1 and/or anti–PD-L1 therapies, demonstrating that the presence of lymphocytes at cancer sites is the basis for effective immunotherapy with ICIs.

#### Future prospects

Currently, the testing of PDL1 expression in the tumor microenvironment [5] and testing for the presence of mismatched repair deficiency in tumor cells [103], which correlates with microsatellite instability and tumor mutational burden, have been approved by the FDA for guidance of ICI therapy in clinics. However, PDL1 expression is often highly heterogeneous within a tumor and often in disagreement between the primary tumor and the metastatic lesions [104–106], which poses a challenge in using the information obtained from analysis of single small biopsy samples in clinical practice. Knowledge gained from adoptive cell therapy might provide some new ideas in searching for novel predictive biomarkers. For example, it might be interesting to determine whether levels or dynamic changes of CD8^+^PD-1^+^ T cells in peripheral blood [80, 107] are associated with treatment responses to ICIs.

Identification of new therapeutic targets and/or development of new therapeutic agents that modulate immune responses will broaden the applications of immunotherapy for cancers. Similarly, strategies that enhance immunogenicity of tumor cells or attract immune cells to cancer sites are expected to be effective approaches to overcoming primary resistance. Indeed, locoregional oncolytic virotherapy has been shown to sensitize tumors resistant to anti–PD-1 or anti–PD-L1 therapy preclinically and clinically [99, 100], presumably because locoregional inflammatory signals promote lymphocyte infiltration at cancer sites. Therapeutic approaches that induce immunogenicity of cancer cells, such as inducing immunogenic cell death [108] by radiotherapy [109, 110], chemotherapy [111], or chemoradiotherapy [112], are also expected to attract lymphocytes to cancer sites, thereby sensitizing tumors to ICI therapy. Therefore, combining ICIs with therapies that promote immunogenicity in tumors or attract lymphocytes to cancer sites will likely be further pursued both preclinically and clinically. A recent phase III trial in patients with previously untreated metastatic nonsquamous NSLCL without EGFR or ALK mutations showed that standard chemotherapy plus pembrolizumab resulted in significantly longer OS and PFS than chemotherapy alone [113]. The benefit of adding pembrolizumab was observed in all subgroups, including those with PD-L1 expression of < 1%, demonstrating feasibility of enhancing immunogenicity in tumors via chemotherapy. On the other hand, loss of antigen presentation caused by genetic alterations in genes involved antigen presentations, such as B2M [94] and HLA [84], has been reported in acquired resistance to anticancer immune therapy. Overcoming the resistance caused by a loss of antigen presentation may require strategies that eliminate cancers independent of HLA, such as adoptive cell therapy with NK cells or chimeric antigen receptor T cells. Progress has also been made in the field of adoptive cellular therapy [114]. The knowledge gained from both ICIs and adoptive cell therapy is expected to have a tremendous impact on the clinical practice of immunotherapy for lung cancer.

## Abbreviations

FDA: The United States Food and Drug Administration
GM-CSF: Granulocyte-macrophage colony-stimulating factor
HLA: Human leukocyte antigen
HR: Hazard ratio
ICI: Immune checkpoint inhibitor
IDO: Indoleamine-2,3-dioxygenase
IFN: Interferon
ITT: Intent to treat
NK: Natural killer
NSCLC: Non-small cell lung cancer
ORR: Objective response rate
OS: Overall survival
PD-1: Programmed death 1
PD-L1: Programmed death ligand 1
PFS: Progression-free survival
PS: Proportion score
Sq: Squamous
TCIR: T-cell inhibitory receptor
TIL: Tumor-infiltrating lymphocyte
TIM3: T-cel immunoglobulin mucin-3
TMB: Tumor mutation burden
TNF: Tumor necrosis factor alpha
Treg: T regulatory cell
